# Bio-Specific Au/Fe^3+^ Porous Spongy Nanoclusters for Sensitive SERS Detection of *Escherichia coli* O157:H7

**DOI:** 10.3390/bios11100354

**Published:** 2021-09-24

**Authors:** Yuzhi Li, Fei Gao, Chang Lu, Marie-Laure Fauconnier, Jinkai Zheng

**Affiliations:** 1 Institute of Food Science and Technology, Chinese Academy of Agricultural Sciences, Beijing 100193, China; yuzhili_226@163.com (Y.L.); QXGF00972819@163.com (F.G.); luchang@caas.cn (C.L.); 2 Laboratory of Chemistry of Natural Molecules, Gembloux Agro-Bio Tech, University of Liege, 5030 Gembloux, Belgium; marie-laure.fauconnier@uliege.be

**Keywords:** SERS, *E. coli* O157:H7, detection, Au/Fe^3+^ nanoclusters, biosensor

## Abstract

For sensitive and fast detection of *Escherichia coli* O157:H7, organic and inorganic hybrid Au/Fe^3+^ nanoclusters (NCs) were synthesized for the first time using gold nanoparticles (GNPs), bovine serum albumin, ferric chloride, phosphate-buffered saline, and antibodies. The Au/Fe^3+^ porous spongy NCs with large surface area showed excellent bio-specific capability for *E. coli* O157:H7. GNPs in Au/Fe^3+^ NCs functioned as signal enhancers, significantly increasing the Raman signal via the metathesis reaction product of Prussian blue and obviously improving the detection sensitivity. We combined the novel Au/Fe^3+^ NCs with antibody-modified magnetic nanoparticles to create a biosensor capable of sensitive detection of *E. coli* O157:H7, which showed a good linear response (10^1^ to 10^6^ cfu/mL), high detection sensitivity (2 cfu/mL), and good recovery rate (93.60–97.50%) in spiked food samples. These results make the biosensor well-suited for food safety monitoring. This strategy achieves the goal of sensitive and quantitative detection of *E. coli* O157:H7.

## 1. Introduction

Food safety problems caused by foodborne pathogens have attracted increasing concern globally. *Escherichia coli* O157:H7 is one of the main foodborne pathogens; it passes through the entire food supply chain from farm to table. *Escherichia coli* O157:H7 can cause diarrhea, abdominal pain, and hemolytic-uremic syndrome, which is dangerous to children [1]. According to the World Health Organization, the global incidence of *E. coli* is 2.8 million cases per year. Enterotoxigenic *E. coli* infections caused 51,186 deaths worldwide according to the 2016 Global Burden of Diseases, Injuries, and Risk Factors study [2]. Traditional methods for *E. coli* O157:H7 detection, which include culture plate [3], polymerase chain reaction [4], and enzyme-linked immunosorbent assay [5], have limitations including low sensitivity, the requirement for trained personnel, and/or a long testing time. A sensitive, fast, and specific detection method is urgently needed to avoid *E. coli* O157:H7 infectious outbreaks and improve treatment.

Surface-enhanced Raman scattering (SERS) is a spectroscopic technique based on molecular vibrations, in which Raman signals are greatly enhanced via interaction with rough metal surfaces. The enhancement is due to local surface plasmon resonance, or “hot spots” located at junctions [6,7,8]. SERS-based biosensors have been proven to be powerful tool for pathogen detection due to its precision, non-requirement for tedious sample pretreatment, high sensitivity, and quantitative detection capability [9,10,11,12,13,14]. Gold nanoparticles (GNPs), which are the most commonly used SERS substrate, have the advantages of facile synthesis, stable colloidal properties, and excellent signal enhancement effect [15,16]. To improve sensitivity, GNPs are encapsulated with Raman reporters as SERS tags, which provide strong Raman signals due to plasmonic hot spots at junctions between closely packed GNPs [17]. To improve specificity, GNPs are often linked with biorecognition groups, such as glutaraldehyde, aptamers, or antibodies [11,18,19]. Among these, monoclonal antibodies are mostly commonly used due to their high specificity and uniformity. However, direct Raman signals produced by SERS tags exhibit limited signal enhancement and are easily influenced by the surroundings. Signal amplification is needed to enhance signal strength and detection sensitivity.

The catalytic activity of certain inorganic ions and organic hybrid nanomaterials has the potential for signal enhancement in pathogen detection applications [20]. The inorganic ions of interest include Cu^2+^, Fe^3+^, Ca^2+^, and Au^3+^, which can be fabricated into nanoparticles (NPs) having very large specific surface areas [21,22,23,24]. The organic materials include bovine serum albumin (BSA), enzymes, and antibodies, which are more suited to biological applications such as enhancing enzymatic activity and biorecognition of antibodies [25,26,27]. For example, immune Ca^2+^ nanoclusters (NCs) have been constructed for pathogen detection by an electrochemical biosensor [28], highly fluorescent Au NCs have been synthesized for sensitive detection of *E. coli* [29], and Fe^3+^ NCs have been used for rapid colorimetric detection of *Salmonella* via smart-phone imaging [20]. The ferric ion (Fe^3+^) present in Prussian blue (PB) can function as a highly sensitive and background-free resonant Raman reporter; it displays a strong and sharp single vibrational peak at 2150 cm^−1^ [30]. Furthermore, PB has been assembled onto GNPs to provide SERS tags with a high signal-to-background ratio [30]. We propose that NCs consisting of Fe^3+^ and antibodies might be feasible for *E. coli* O157:H7 SERS detection via the characteristic Raman signal of PB following reaction with Fe^3+^ NCs and biorecognition of antibodies. By adding GNPs to Fe^3+^ NCs, significant Raman signal enhancement by the GNPs could improve detection of *E. coli* O157:H7.

**Figure 1:**
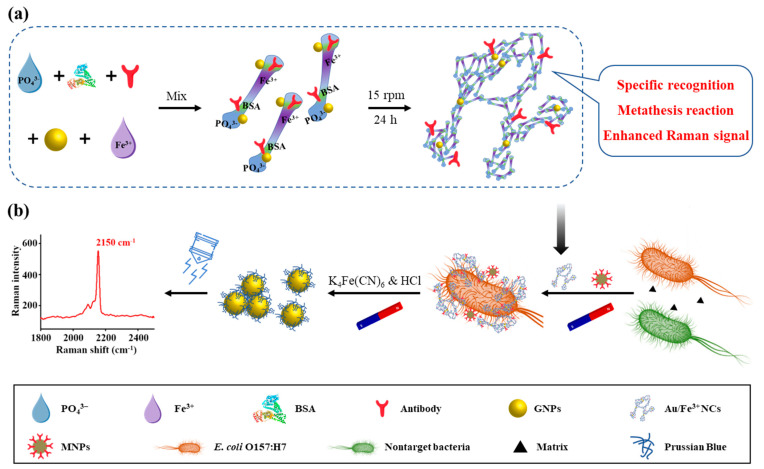
Schematic illustration of the SERS detection biosensor. (**a**) Principle of the one-pot synthesis of Au/Fe^3+^ NCs from PBS, BSA, FeCl_3_, Au nanoparticles, and antibodies and (**b**) separation and detection of *E. coli* O157:H7.

Herein, we describe a sensitive and rapid SERS biosensor for *E. coli* O157:H7 based on novel Au/Fe^3+^ NCs. The Au/Fe^3+^ NCs, which were synthesized using inorganic components (PBS, GNPs, Fe^3+^) and organic components (BSA, antibodies complementary to *E. coli* O157:H7), had a porous spongy structure with high specific surface area (Figure 1a). Reaction with Fe^3+^ of the PB realized Raman signals, the GNPs provided stable and strong signal enhancement, and antibodies offered biorecognition of *E. coli* O157:H7. The Au/Fe^3+^ NCs specifically labeled target pathogens to form sandwich complexes with antibody-modified magnetic nanoparticles (MNPs), thereby realizing sensitive SERS detection of the pathogens via indirect Raman signal from *E. coli* O157:H7, but from Prussian blue (Figure 1b). This biosensor, benefiting from the novel Au/Fe^3+^ NCs, achieved the goal of sensitive and quantitative detection of *E. coli* O157:H7. This biosensor may become an effective tool for the detection of foodborne pathogens.

## 2. Materials and Methods

### 2.1. Materials and Reagents

Chloroauric acid trihydrate (HAuCl_4_), trisodium citrate, BSA, and ferric chloride (FeCl_3_) were purchased from Sigma–Aldrich (St. Louis, MO, USA). Phosphate-buffered saline (PBS; pH 7.4, 10 mM) was obtained from Gibco (Beijing, China). Streptavidin-modified MNPs of 150-nm diameter were purchased from Ocean Nanotech (Dunedin, FL, USA). Luria–Bertani medium (LB), agar and alkaline peptone water medium were obtained from Aoboxing Biotech (Beijing, China). *Escherichia coli* O157:H7 (ATCC 43888), *E. coli* (ATCC 25922), *Salmonella typhimurium* (ATCC 14028), *Staphylococcus aureus* (ATCC 25923) and *Vibrio parahemolyticus* (ATCC 17802) were purchased from Solarbio Life Sciences (Beijing, China). Potassium ferrocyanide and hydrochloric acid were obtained from Sinopharm (Shanghai, China). Rabbit antibody against *E. coli* O157:H7 was purchased from Meridian Life Science (Memphis, TN, USA). A Long-arm Biotin Labeling Kit from Elabscience Biotechnology (Wuhan, China) was used for the modification of antibodies. Ultrapure water was prepared using a Milli-Q system (Bedford, MA, USA).

### 2.2. Preparation of Novel Au/Fe^3+^ Nanoclusters

The novel Au/Fe^3+^ NCs were fabricated via a one-pot synthesis method (Figure 1). Gold NPs were prepared via a chemical reduction method, as previously reported [15]. Briefly, 1 mL of HAuCl_4_ (1%, *w*/*v*) was dissolved in 100 mL of ultrapure water and boiled. Then, 4 mL of sodium citrate (1%, *w*/*v*) was rapidly added under magnetic stirring and the solution was boiled for 15 min until it turned dark red. The prepared GNPs were purified by centrifugation (8000× *g*, 10 min). The Au/Fe^3+^ NCs were prepared as follows. First, 30 µL of 1 mg/mL BSA and 2 µL of 1 mg/mL antibodies were dissolved in 10 mM PBS buffer (pH 7.4). The synthesized GNPs (200 µL; OD 0.5) were mixed with the solution and shaken vigorously, followed by addition of 100 µL of 100 mM FeCl_3_. The mixture was then incubated at room temperature for 24 h at 15 rpm, centrifuged at 5000× *g* for 5 min to remove the supernatant, and finally resuspended in ultrapure water for later use.

### 2.3. Characterization of Au/Fe^3+^ Nanoclusters

Transmission electron microscopy (TEM; model HT7700; Hitachi, Tokyo, Japan) was used to characterize nanostructure morphology. High-resolution TEM (HRTEM) and energy-dispersive spectrometry (EDS) were conducted for studying elemental distributions using a JEM-2100F microscope (JEOL, Tokyo, Japan) at 200 kV accelerating voltage. A UV-1780 spectrometer (Shimadzu, Kyoto, Japan) was used to record Ultraviolet-visible (UV-vis) spectra. A confocal Raman microscope (JY H-800; Horiba, Kyoto, Japan) equipped with a 633-nm He–Ne excitation laser was used for SERS spectrum collection. Raman peaks range from 1800 to 2500 cm^−1^ was collected under 12-mW laser power with 10 s integration time. The Raman signal intensity was measured at five random spots.

### 2.4. Separation and Detection of Bacteria

Fe_3_O_4_-antibody capture probes were prepared by conjugating monoclonal antibodies onto MNPs according to previously method [19]. Then, 30 µL of antibody-modified MNPs was incubated with different concentrations of *Escherichia coli* O157:H7 (10^1^ to 10^8^) for 15 min at 15 rpm to form MNP–bacteria complexes, and washed twice by magnetically separation. Then, 30 µL of the prepared Au/Fe^3+^ NCs was added, followed by 15-min incubation at 15 rpm. The capture probe–pathogen–Au/Fe^3+^ NCs sandwich complexes were collected and washed twice with PBS under a magnetic field. Then, 10 µL of 6% HCl was added to the collected sandwich complexes and vortexed to mix. The MNPs were separated and removed. Potassium ferrocyanide (6 µL of 100 mM) was added and the solution was mixed to create PB for later SERS experiments.

### 2.5. Optimization of the Au/Fe^3+^ Nanoclusters-Based Biosensor

Different volumes of Fe^3+^ (25, 50, 75, 100, 125, and 150 µL) and GNPs (50, 100, 150, 200, 250, and 300 µL) were tested to optimize the conditions for fabrication of Au/Fe^3+^ NCs. During the detection procedure, HCl was used to release Fe^3+^ from Au/Fe^3+^ NCs for subsequent metathesis reaction. The volume of HCl (0, 2.5, 5, 7.5, 10, 12.5, and 15 µL) was also optimized to obtain the strong Raman signal intensity of the fabricated Au/Fe^3+^ NCs and maximize the detection sensitivity. Then, we determined the optimal concentration of Au/Fe^3+^ NCs by adding various concentrations (15, 20, 25, 30, 35, and 40 nΜ) of Au/Fe^3+^ NCs to 10^5^ cfu/mL of *E. Coli* O157:H7.

### 2.6. Performance Evaluation of the Biosensor

To measure the sensitivity of the biosensor, different concentrations of *E. coli* O157:H7 10^1^–10^8^ cfu/mL) were detected and the limit of detection (LOD) was calculated. To evaluate the specificity of the biosensor, different kinds of bacteria including *E. coli*, *S. typhimurium*, *S. aureus*, and *V. parahemolyticus* were used. In addition, 18 random samples from the same *E. coli* O157:H7 sample at 10^2^ cfu/mL were selected to measure the reproducibility of the biosensor.

### 2.7. Analyses of Food Samples

Samples of tap water, lettuce, and chicken were used for realistic detection of *E. coli* O157:H7 and the preparation of samples was the same as reported previously, with minor modifications [15]. The recovery and accuracy of this method was calculated by compared to the traditional plate counting method.

### 2.8. Data Analyses

LabSpec software and TQ Analyst software (v. 8.0; Thermo Fisher Scientific, Waltham, MA, USA) were used to record and analyze the SERS spectra. All analyses of SERS detection were performed at least five times, and each plate-counting determination was done in triplicate. Origin 8.0 software (OriginLab, Northampton, MA, USA) was used to draw the figures.

## 3. Results and Discussion

### 3.1. Fabrication and Characterization of Au/Fe^3+^ Porous Spongy Nanoclusters

**Figure 2:**
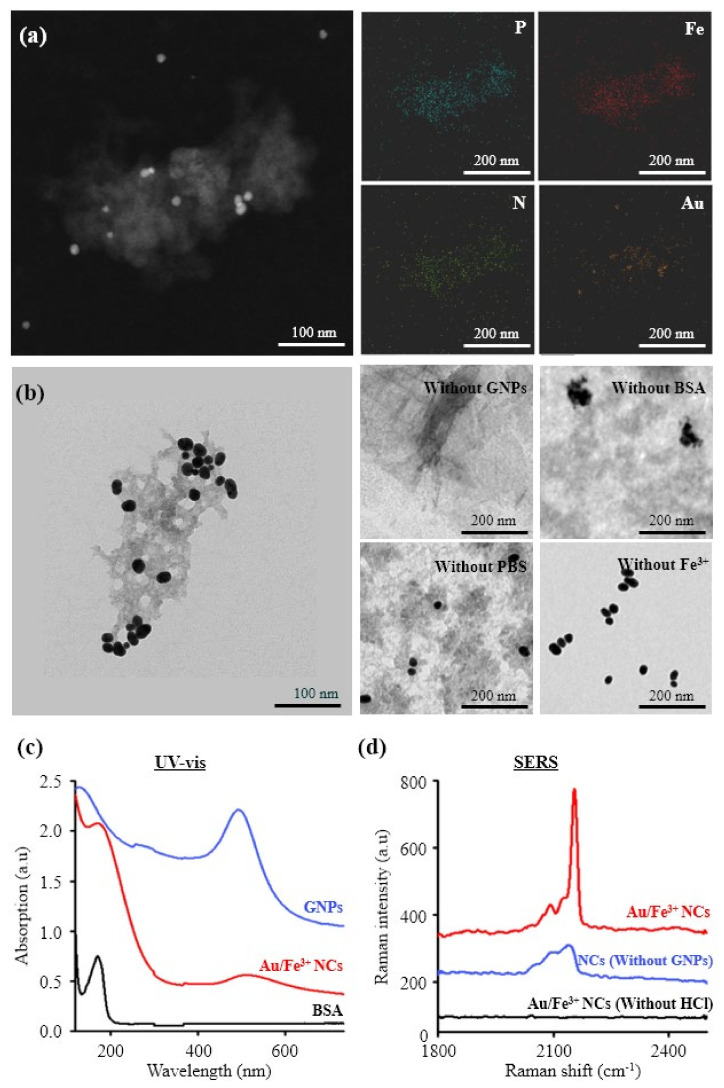
Characterization of Au/Fe^3+^ NCs. (**a**) HRTEM images and energy dispersive spectrometry images of Au/Fe^3+^ NCs; P = PBS, Fe = FeCl_3_, N = BSA and antibody, Au = gold nanoparticle. (**b**) TEM images of Au/Fe^3+^ NCs and synthesized products with one reagent missing. (**c**) Ultraviolet absorption spectra of Au nanoparticles, BSA, and Au/Fe^3+^ NCs. (**d**) Raman enhancement by Au/Fe^3+^ NCs after metathesis reaction.

The Au/Fe^3+^ NCs, which are critical to the performance of the biosensor, were characterized by HRTEM, EDS, TEM, UV–vis absorption spectroscopy and SERS. Figure 2a shows that the ~300-nm diameter prepared Au/Fe^3+^ NCs had a porous spongy structure and GNPs were uniformly distributed on them. The porous spongy structure provides advantages for the better recognition of antibodies due to the big specific area and makes it easier for the release of iron ions for later PB reaction, both contributing to the sensitive and specific detection of *E. coli* O157:H7. The EDS characterization demonstrated successful fabrication of Au/Fe^3+^ NCs by PBS (element P), BSA and antibodies (element N), FeCl_3_ (element Fe), and GNPs (element Au), which suggested the necessity of four kinds of reagents for synthesis of the porous spongy structure of the Au/Fe^3+^ NCs.

To verify this assumption, we synthesized Au/Fe^3+^ NCs with one reagent missing (Figure 2b). In the absence of GNPs, the synthesized NCs were flake shaped. In the absence of BSA, the GNPs agglomerated during the synthesis process due to a lack of protection from ions in the PBS solution. In the absence of PBS, which could function as a skeleton during the Au/Fe^3+^ NC synthesis [23], the BSA could not complex well, and a loose flocculant structure resulted. In the absence of FeCl_3_, which was considered as one type of bonding point of Au/Fe^3+^ NCs, the BSA complex reaction was also affected and less BSA adsorbed on the surface of the GNPs. These results confirmed the necessity of all of these reagents for successful Au/Fe^3+^ NCs synthesis.

The Au/Fe^3+^ NCs also exhibited strong Raman signal enhancement, presumably due to the presence of the GNPs that enhanced the PB Raman signal, which formed via the release of Fe^3+^ by HCl treatment and Fe^2+^ in potassium ferrocyanide. Figure 2d shows that the designed Au/Fe^3+^ NCs displayed the characteristic strong Raman peak of PB at 2150 cm^−1^. The signal intensity was related to the Fe^3+^ and GNPs contents of the NCs. When no HCl was present to release Fe^3+^ from the Au/Fe^3+^ NCs, no PB was generated and thus no Raman signal appeared. If there were no GNPs in the NCs, PB could still be produced due to the Fe^3+^ present in the flake-shaped product, but the Raman signal intensity was much weaker. This result confirmed our hypothesis that the GNPs functioned as signal enhancers. The strong Raman signal intensity greatly improved the detection sensitivity of the biosensor.

### 3.2. Formation Mechanism of Au/Fe^3+^ Nanoclusters

**Figure 3:**
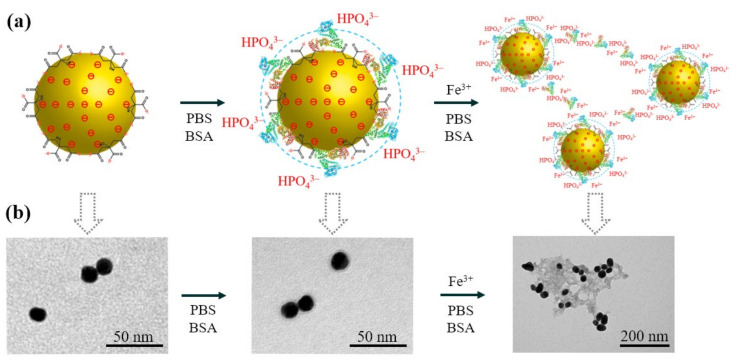
Hypothesis and verification of the synthesis process of Au/Fe^3+^ NCs. (**a**) Schematic illustration of the proposed synthesis of Au/Fe^3+^ NCs and (**b**) scanning electron microscope images of the steps.

The GNPs were well-dispersed in the absence of PBS or FeCl_3_, but were aggregated in the BSA-free sample. Our proposed synthetic mechanism for the one-pot synthesis process (Figure 3a) was supported by scanning electron microscope observations (Figure 3b). Citrate anions, functioning as an electrostatic stabilizing agent, covered the surface of the GNPs after synthesis [31]. The negative surface charge of the citrate layer provided mutual repulsion between neighboring GNPs and offered electrostatic stabilization. The GNPs aggregated in a salt solution due to destruction of the citrate layer by the positive ions. When GNPs were added to a BSA solution in PBS, positively charged groups of BSA adsorbed on the surface of GNPs, which protected the GNPs from the positive ions of PBS, thereby preventing aggregation. Previous quartz crystal microbalance with dissipation monitoring (QCM-D) and ζ-potential measurements suggested that the dominant association of BSA with citrate-coated NPs was electrostatic [32]. The electrostatic interactions between citrate and lysine on the protein surface were of the carboxylate–ammonium type. Studies of the binding of BSA to Au surfaces revealed nonspecific binding to self-assembled monolayers with NH_3_^+^ [33,34]. Besides the support provided by strong electrostatic binding, the steric interactions between protein and citrate on the surface layer of GNPs could provide greater stability than pure electrostatic interactions, due to the reduction of entropy and loss of solvation enthalpy resulting from the interactions of protein side chains or domains.

The Au/Fe^3+^ NCs formed as Fe^3+^ and more PBS and BSA were added. Interaction between the protein and inorganic ions then led to the growth of micrometer-sized particles having nanoscale features [23]. Previous research suggested that coordination between the protein and Fe^3+^ might be the main driver of nanoflower formation. The N atoms of the amide groups in the protein backbone and some amino acid residues, such as histidine, could form complexes with Fe^3+^ [35,36]. Figure 3b presents the TEM results. The GNPs of ~15-nm diameter were well dispersed. Added BSA adsorbed on the surface of GNPs, and added Fe^3+^ formed Au/Fe^3+^ NCs having a porous spongy structure. These findings are in good agreement with previous research concerning the mechanism of formation of the organic–inorganic material.

### 3.3. Optimization of Au/Fe^3+^ NCs-Based SERS Biosensor

**Figure 4:**
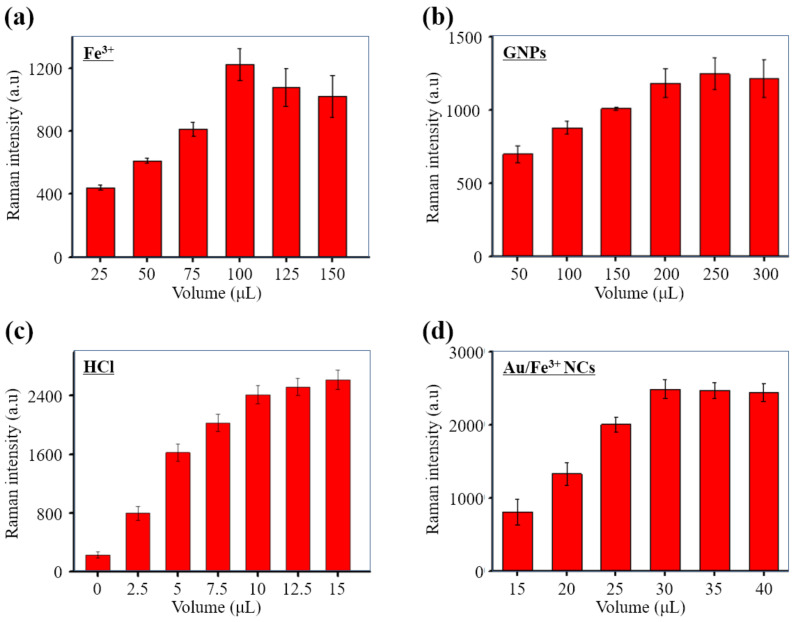
Optimization of the SERS biosensor. Effects of (**a**) Fe^3+^ concentration on Au/Fe^3+^ NCs, (**b**) Au nanoparticle concentration on Au/Fe^3+^ NCs, (**c**) HCl concentration on *E. coli* O157:H7 detection, and (**d**) the volume of Au/Fe^3+^ NCs on *E. coli* O157:H7 detection.

The Fe^3+^ content of Au/Fe^3+^ NCs enabled the production of PB, with its single intense Raman signal. Meanwhile, the GNPs in Au/Fe^3+^ NCs were important for SERS signal enhancement. Thus, the volumes of Fe^3+^ and GNPs were tested to optimize the conditions for fabrication of the Au/Fe^3+^ NCs. With increasing amounts of Fe^3+^ or GNPs, the Raman signal intensity of the SERS tags increased, reached an optimum, and then decreased (Figure 4a,b). To facilitate sensitive detection of *E. coli* O157:H7, the optimal volumes of Fe^3+^ and GNPs for the one-pot syntheses of the Au/Fe^3+^ NCs were 100 and 200 μL, respectively.

During the detection procedure, HCl was used to release Fe^3+^ from the Au/Fe^3+^ NCs for subsequent metathesis reaction to produce PB. The structure of NCs was broken after adding HCl, and GNPs were released. The BSA on surface of GNPs might be damaged under acidic condition. Therefore, the new produced PB could attached nearly on GNPs and generate localized surface plasmon resonance during SERS measurement. On the other hand, the stability of released GNPs was disappeared, and GNPs gathered which could contribute to plasmonic hot spots during SERS measurement. To optimize the volume of HCl, various volumes were evaluated, and the resulting Raman signal intensity was monitored to maximize the detection sensitivity. Figure 4c shows that the Raman signal intensity increased as the volume increased from 0 to 15 μL. The final choice of 10 μL provided the highest signal intensity and avoided reduction of the MNPs.

To determine the optimal concentration of Au/Fe^3+^ NCs in the biosensor system, various concentrations of Au/Fe^3+^ NCs were added to a 10^5^ cfu/mL suspension of *E. coli* O157:H7. The strongest characteristic Raman signal for the sandwich complexes was obtained when the volume of Au/Fe^3+^ NCs added was 30 μL (Figure 4d). This result also demonstrated specific binding between *E. coli* O157:H7 and Au/Fe^3+^ NCs.

### 3.4. High-Performance SERS Detection of E. coli O157:H7

**Figure 5:**
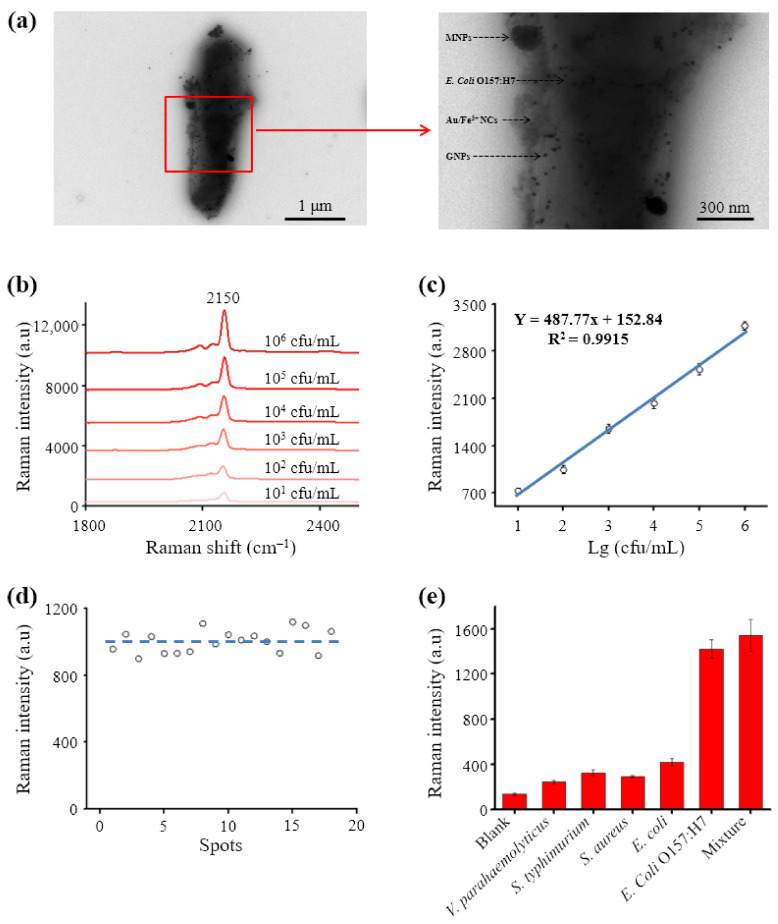
Bio-specificity of the capture probes and Au/Fe^3+^ NCs. (**a**) TEM image of capture probe–*E. coli* O157:H7–Au/Fe^3+^ NC sandwich complexes and corresponding magnified image obtained from the square area, (**b**) SERS spectrum for a series of concentrations of *E. coli*. O157:H7, (**c**) linear correlation between the logarithms of *E. coli*. O157:H7 concentration and SERS peak intensities at 2150 cm^−1^, (**d**) Raman intensities of 18 separately measured random samples from a 10^2^ cfu/mL suspension of *E. coli*. O157:H7, and (**e**) Raman intensities for *E. coli* O157:H7, three interfering bacteria, and their mixture.

The formation of capture probe–pathogen–Au/Fe^3+^ NCs sandwich complexes was also confirmed by TEM imaging (Figure 5a). The magnified TEM images of the square area showed that the MNPs and Au/Fe^3+^ NCs were linked to the surface of *E. coli* O157:H7. The stability and specificity of the capture probes contributed to the sensitive detection of the pathogens. The capture capability of the antibody-modified MNPs was confirmed by traditional plate counting, according to previous work [19]. The detection of *E. coli* O157:H7 was carried out under the optimized conditions.

The favorable linear concentration range was explored using gradient concentrations (10^1^–10^8^ cfu/mL) of *E. coli* O157:H7 and monitoring the Raman peak of PB at 2150 cm^−1^ (Figure 5b). The Raman signal intensity grew rapidly with increasing pathogen concentration from 10^1^ to 10^6^ cfu/mL. With the increasing amount of *E. coli* O157:H7, more NCs containing Fe^3+^ and Au NPs could be separated via “sandwich” structure. Correspondingly, much more PB will produce to generate higher Raman signal. There was a strong linear correlation between the intensity of the Raman signal and the logarithm of the concentration of *E. coli* O157:H7 (y = 487.77x + 152.84, R^2^ = 0.9915), with an LOD value of 2 cfu/mL (Figure 5c). The limit of detection (LOD) was calculated according to the formula LOD = 3N/S, where N is the standard deviation of measurements of a blank sample and S is the slope of the standard curve. These results demonstrate that the biosensor was sufficiently sensitive for quantitative detection of *E. coli* O157:H7. The detection sensitivity was determined by the high specificity of antibodies. Antibodies on MNPs and NCs can recognize the respective target pathogen, and thus the dual recognition ensures detection specificity. This behavior was attributed to the high specificity of the capture probe and the synthesized Au/Fe^3+^ NCs, and the strong signal strength of the Raman peak.

Good reproducibility was verified by taking 18 random samples from a 10^2^ cfu/mL suspension of *E. coli* O157:H7; an RSD of 6.94% was obtained (Figure 5d). To demonstrate the specificity of the biosensor, *E. coli*, *S. typhimurium*, *S. aureus*, and *V. parahemolyticus* were used as interference bacteria. The signal intensities with *E. coli* O157:H7 were almost five-fold stronger than those of the nontarget pathogens (Figure 5e). Furthermore, *E. coli* O157:H7 was precisely detected in mixed samples, which was attributed to the high specific affinity of the Au/Fe^3+^ NCs and capture probes for the target pathogen. Good reproducibility and specificity confirmed the reliability of our detection method.

To further investigate the applicability of the SERS biosensor for *E. coli* O157:H7 in practical use, tap water, lettuce, and chicken were used as models, and our detection results were compared to the classic plate-counting method as a standard [15]. Table 1 shows that the recovery rate for *E. coli* O157:H7 ranged from 93.60% to 97.50%, indicating applicability and good accuracy of the proposed biosensor for the quantification of *E. coli* O157:H7 in food samples. These results further demonstrate the biorecognition specificity of Au/Fe^3+^ NCs. Compared to other reports concerning the use of NCs for pathogen detection, for which fluorescent and colorimetric methods are commonly used [20,24], our SERS biosensor method is applicable and sensitive for pathogen detection (Table 2). The lower LOD values and wider linear range observed with our biosensor confirmed its outstanding quantitative performance, which is attributed to the novel Au/Fe^3+^ NCs for the following reasons: (1) high specificity of antibodies on the surface of porous spongy Au/Fe^3+^ NCs due to big specific area; (2) the background-free and sharp single characteristic Raman peak of PB produced by Fe^3+^; and (3) the strong Raman signal enhancement by GNPs in Au/Fe^3+^ NCs. In addition, antibodies to other kinds of target (e.g., *S. typhimurium*, cancer cells or inflammatory factors) could be used for magnetic nanoparticles modification and nanocluster fabrication. Correspondingly, the SERS biosensor detection of these targets could be realized. Furthermore, in conjunction with portable Raman equipment, there is the possibility of on-site pathogen detection.

## 4. Conclusions

Sensitive SERS detection of *E. coli* O157:H7 was achieved using a biosensor containing novel Au/Fe^3+^ NCs, which were synthesized from GNPs, BSA, FeCl_3_, PBS, and antibodies. The porous spongy Au/Fe^3+^ NCs increased the Raman signal intensity of PB, which was formed from the release of Fe^3+^ after treatment with HCl, and from Fe^2+^ in potassium ferrocyanide. The Raman signal from Prussian blue rather than from *E. coli* O157:H7 was further enhanced by the GNPs. The Au/Fe^3+^ NCs also exhibited specific biorecognition capacity due to the attached antibodies, which boosted detection sensitivity. Specific separation and enrichment of target pathogens was facilitated by monoclonal antibody-modified MNPs. This biosensor was characterized by a good quantitative response between the Raman signal intensity and logarithm of the concentration of *E. coli* O157:H7 over the range of 10^1^–10^6^ cfu/mL, with an LOD value of 2 cfu/mL. The recovery rates from three spiked food samples ranged from 93.60% to 97.50%, demonstrating the potential of the biosensor method for effective detection of *E. coli* O157:H7 and early screening of contaminated food. This biosensor, benefiting from the porous spongy structured Au/Fe^3+^ NCs, may become a universal tool for effective, sensitive, and specific detection of various foodborne pathogens.

## Figures and Tables

**Table 1:** Comparison of the detection of *E. coli* O157:H7 in three real samples between the surface-enhanced Raman scattering biosensor and classic plate counting.

	Spiked Concentration (cfu/mL)	Plate Counting Method (cfu/mL)	This Method (cfu/mL)	Recovery Rate (%)
Tap water	1000	906 ± 70	968 ± 89	93.60
Lettuce	1500	1486 ± 30	1418 ± 113	95.25
Chicken	1500	1633 ± 31	1674 ± 83	97.50

**Table 2:** Comparison of the detection method, target pathogens, limit of detection (LOD), and detection time of this study with related detection material in other reports.

Methods	Materials	Target	LOD (cfu/mL)	Detection Time	Ref.
SERS	AuMNPs core/shell nanocomposites	*S. aureus*	10	~120 min	[13]
SERS	Gold nanoparticles	*S. typhimurium* & *S. aureus*	15 & 35	~150 min	[15]
Electrochemical biosensor	GOx&HRP-Cu_3_(PO_4_)_2_ hybrid nanoflowers	*E. coli*	1	~140 min	[7]
Hue-saturation-lightness color space	Fe-nanoclusters	*S. typhimurium*	14	—	[20]
Hue-saturation-lightness color space	Immune GOx–nanoclusters	*S. typhimurium*	16	—	[24]
SERS	Nanocluster	*E. coli* O157:H7	2	~30 min	This study

## Data Availability

Not applicable.
